# How can culture and compassion shape better pediatric palliative care for Filipino families?

**DOI:** 10.1017/S1478951525100692

**Published:** 2025-09-11

**Authors:** Jose Eric Mella Lacsa

**Affiliations:** Theology and Religious Education, De La Salle University, Manila, Philippines

Dear Editor,

The recent study by Stößlein et al., ‘I’m not a physician, but I’m the expert for my child’ (Stößlein et al. 2025) offers valuable insight into the experiences of parents caring for children with life-limiting conditions (LLCs) in inpatient settings. Their findings, highlighting parents’ emotional burdens, unmet psychosocial needs, and the undervaluing of their caregiving expertise, resonate far beyond the European context. In countries like the Philippines, where families play a central role in healthcare, these insights take on even greater significance.

Filipino parents are not only caregivers but also case managers, emotional anchors, and medical aides. Due to gaps in the healthcare system, especially in pediatric palliative services, parents are often left to navigate complex medical care at home with little professional support (Ho et al. 2024). In public hospitals, where resources are stretched thin and provider–patient ratios are low, parents’ voices can be easily overlooked, even though they bring invaluable knowledge from years of hands-on experience.

While the Philippines shares with other countries the need to strengthen family-centered care, it also presents unique cultural dimensions. Family involvement in medical decision-making is deep-rooted, but hospital hierarchies and time-pressured environments may limit meaningful collaboration (Jandt 2021). There is a critical need to shift from a model of compliance to one of partnership, where parents are seen not merely as attendants, but as co-partners in the care team.

Adapting the recommendations of Stößlein et al. to the Philippine setting means investing in systems that acknowledge parental expertise and support their emotional and spiritual well-being. Psychosocial care remains limited in many hospitals, especially for families from rural or underserved areas (Mental Health in Rural Philippines: Overcoming Barriers to Access, 2023). A more holistic approach, integrating mental health support, spiritual care, and community-based resources, would go far in easing the weight many parents carry silently.

Importantly, communication remains a cornerstone. Many Filipino families speak regional languages or dialects, and communication around end-of-life issues must be handled with sensitivity, clarity, and cultural awareness (Stanford University School of Medicine, n.d.). Training programs for healthcare providers can help cultivate this sensitivity, while also encouraging practices that empower families to actively participate in care planning.

What Stößlein et al. underscore, namely, that parents must be supported as equal partners in care, is not just good practice, but an ethical imperative. For the Philippines, and many countries in the Global South, it also presents an opportunity: to build models of pediatric palliative care that are both clinically sound and culturally grounded. By centering parents, especially mothers and fathers who navigate illness with quiet resilience, we take a meaningful step toward more compassionate, inclusive, and responsive palliative care systems.
